# Supplementary figures and images for: Color Quest: An interactive tool for exploring color palettes and enhancing accessibility in data visualization
Source: PLoS One. 2024 Mar 19;19(3):e0290923. doi: 10.1371/journal.pone.0290923 (PMC10950247; doi:10.1371/journal.pone.0290923)

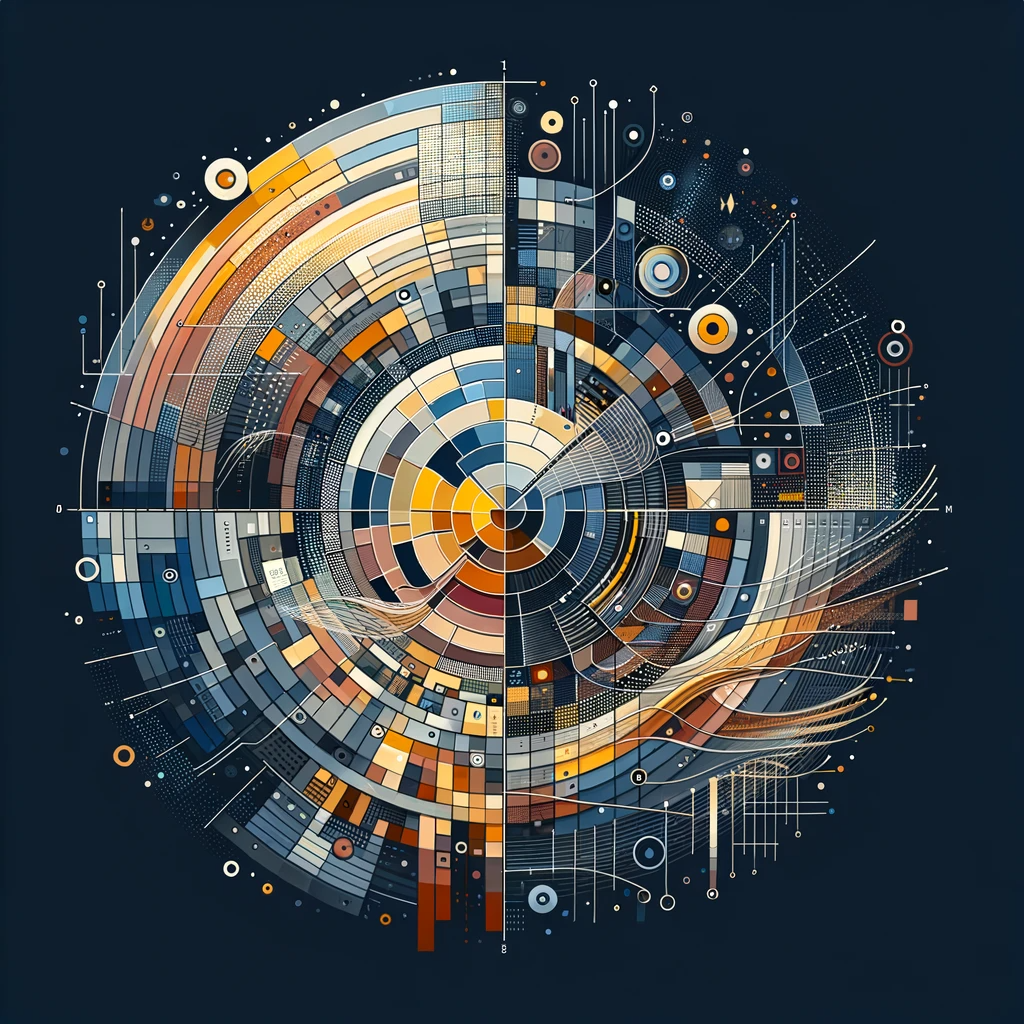

Supplement: S1 File — (PNG) [file pone.0290923.s001.png]
