# Supplementary material for: Color Quest: An interactive tool for exploring color palettes and enhancing accessibility in data visualization
Source: PLoS One. 2024 Mar 19;19(3):e0290923. doi: 10.1371/journal.pone.0290923 (PMC10950247; doi:10.1371/journal.pone.0290923)
Supplement: Attachment — Submitted filename: RESPONSE to reviewers.docx [file pone.0290923.s003.docx]

**Reviewer #1**: Authors of this document asserted an Interactive Tool for Exploring Color Palettes and claiming that this can enhance Accessibility in Data Visualization.

This paper has a good structure with a good description about the proposed solution which looks promising but it doesn’t provide any examples of using it. References are from reputable publishers and also up-to-date.

No literature review was included, which was intended to offer a thorough grasp of the research problem and the various existing or similar solutions. This paper also lacks both metrics for assessing the proposed solution and a verified analysis of its efficacy.

REPLY: Thank you very much for taking the time to go through my manuscript.

Thank you in particular for pointing out the absence of a formal literature review. I acknowledge its importance in establishing the context and foundation for this work. In my efforts to maintain the brevity of this short communication, I initially omitted a detailed review. However, recognizing its value, I have now incorporated a concise mention of a recent literature review on the topic (Stoelzle and Stein 2021). Their comprehensive review critically assessed the use and implications of the color maps and plots in scientific visualizations. By referencing this recent and extensive review, I think I have now better established the context without being redundant (it’s a recent and well done, while addressing your concerns.

With respect to the lack of metrics to assess the tool’s efficacy, I would like to emphasize that the current manuscript is a short communication, primarily focused on introducing the tool to the community. Given the constraints of this format, an exhaustive validation, although valuable, would be beyond the scope of this manuscript. However, recognizing the merit of your feedback and in the spirit of ensuring my tool's utility, I have implemented a user feedback mechanism within the app. This will allow users to provide insights into the tool's effectiveness, usability, and potential areas for improvement. Initial anecdotal evidence from this feedback has been positive and suggests that the tool is meeting its intended goals. In the revised manuscript, I have incorporated a section detailing this user feedback mechanism. I believe this strikes a balance between the constraints of a short communication and the need to demonstrate the tool's practical utility.

**Reviewer #2**: The manuscript introduces "Color Quest", an interactive tool developed to address accessibility issues in data visualization, specifically considering individuals with color vision deficiencies. This is a significant contribution, given the growing emphasis on inclusivity and accessibility in scientific communication. Overall, the manuscript is well-structured and written clearly. The abstract succinctly summarizes the paper's main points and provides a clear understanding of the tool's purpose and potential impact.

REPLY: Thanks a lot for the positive feedback. Please see my replies to specific comments.

Specific Comments:

Line 5: "Glsagow, UK" seems to be a typo. It should be corrected to "Glasgow, UK".

REPLY: Thanks! Now corrected.

Lines 12-13: You mentioned the ability of the tool to visualize color palettes on custom images. Including a brief mention of the feature allowing users to upload their images might provide a more rounded view of the app’s capabilities in the abstract.

REPLY: Thanks for pointing out. I have now added this sentence in the abstract: “. In addition, it enables users to visualize palettes on their own custom-uploaded images.”.

Line 66: The connection between the limitation with black-and-white printers and the black and white option in the tool could be clarified further.

REPLY: Now further clarified by adding “[…] to ensure visuals remain comprehensible even in grayscale print”

Lines 78-79: Mentioning that the code is open-source and available at GitHub is a great point. It would be good to also state if any documentation or community guidelines are available for potential contributors.

REPLY: Thanks for the suggestion, I have now added more documentation on github and expanded the paragraph in the manuscript: “Both the code and its documentation can be found in the supplementary material and are continuously updated on my GitHub repository: https://github.com/lucanelli/colorquest. The full code is available in the supplementary material, and the most updated version is freely available at the author’s github repository at https://github.com/lucanelli/colorquest. On GitHub, users will find detailed documentation, step-by-step instructions, explanations of code functions, and use-case examples.”. In addition, I have implemented a user feedback mechanism within the app. This will allow users to provide insights into the tool's effectiveness, usability, and potential areas for improvement. In the revised manuscript, I have incorporated a section detailing this user feedback mechanism and highlighted some preliminary findings from the user community.

Lines 80-95: The discussion highlights the potential impacts of the tool very well. Adding a glimpse of future developments or enhancements could add more depth to this section. E.g., mentioning a few specific examples or case studies where the tool could be beneficial might give a more grounded view of its potential applications.

REPLY: Great advice, thanks. I have now expanded this section with practical examples and future directions: “For instance, in medicine, Color Quest can be helpful in future genetic research studies, ensuring that subtle genetic variations visualized are discernible by researchers across the spectrum of color vision. In epidemiology, should a future outbreak arise where data visualization plays a critical role in decision-making, Color Quest could be instrumental in crafting universally comprehensible color-coded infection rate maps, facilitating more informed and rapid response strategies. Similarly, in ecology and biodiversity conservation Color Quest may play a transformative role by ensuring that maps detailing biodiversity hotspots or environmental degradation areas are accessible to policymakers and the general public, irrespective of their color vision capabilities”.

Sincerely,

**Luca Nelli (PhD)**

School of Biodiversity, One Health & Veterinary Medicine

University of Glasgow

Room 303, Graham Kerr Building

Glasgow, G12 8QQ
